# Supplementary figures and images for: Neuromuscular denervation and deafferentation but not motor neuron death are disease features in the Smn2B/- mouse model of SMA
Source: PLoS One. 2022 Aug 1;17(8):e0267990. doi: 10.1371/journal.pone.0267990 (PMC9342749; doi:10.1371/journal.pone.0267990)

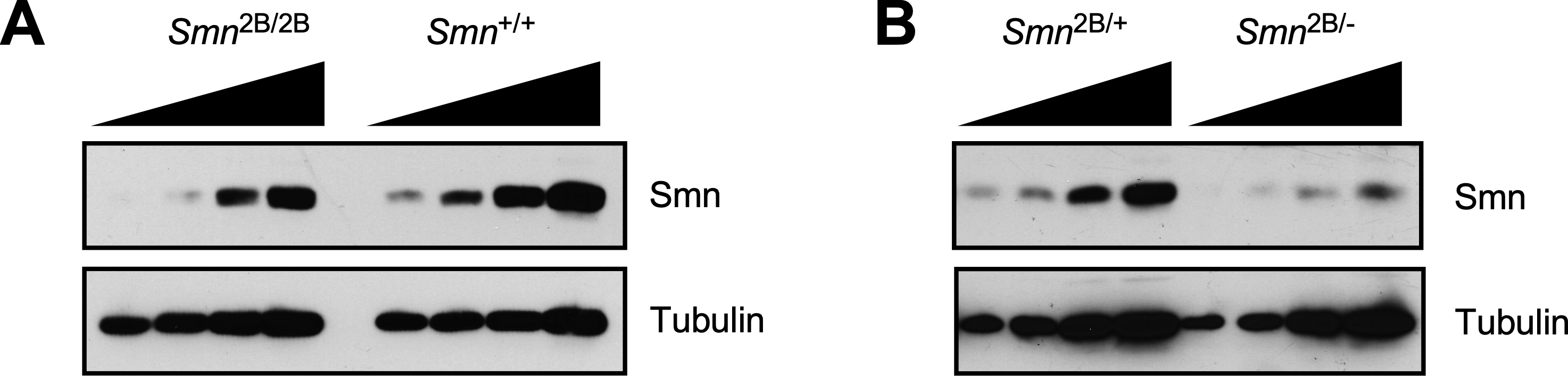

Supplement: S1 Fig — (A) Western blot analysis of Smn levels in the spinal cord from Smn+/+ (wild type) and Smn2B/2B mice at P16. (B) Western blot analysis of Smn levels in the spinal cord from Smn2B/+ an Smn2B/- mice at P16. Two-fold serial dilutions of equal amounts of extracts are shown. Tubulin was probed as a loading control. (TIF) [file pone.0267990.s002.tif]

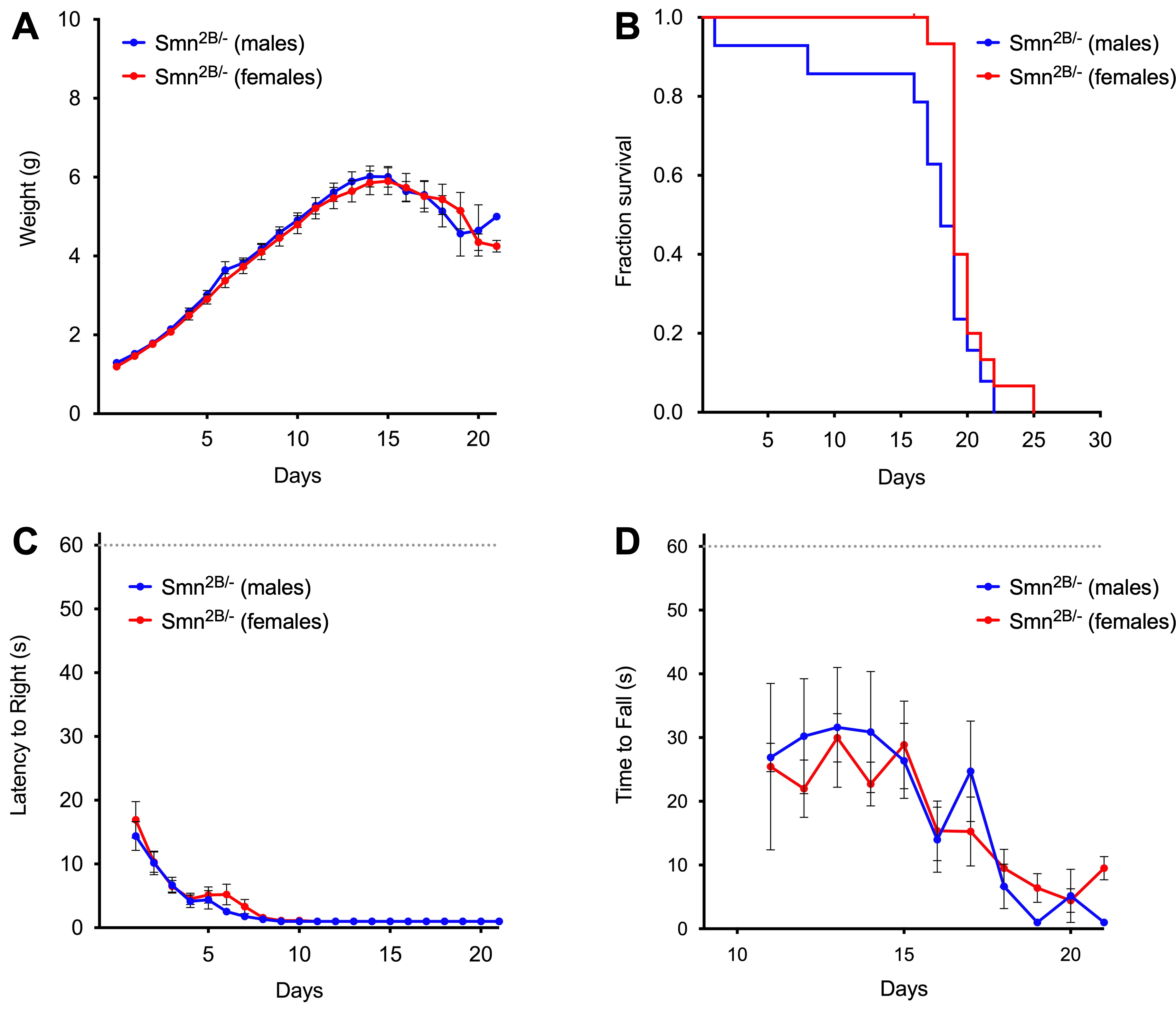

Supplement: S2 Fig — (A) Body weight of male (n = 14) and female (n = 17) Smn2B/- SMA mice. Data represent mean and SEM. Statistics were performed with two-way ANOVA and Bonferroni’s multiple comparison test. Not Significant. (B) Kaplan-Meier survival curves from the same experimental groups as in (A). Statistics were performed with Log-rank (Mantel-Cox) test. Not Significant. (C) Righting time from the same experimental groups shown in (A). Data represent mean and SEM. Statistics were performed with two-way ANOVA and Bonferroni’s multiple comparison test. Not Significant. (D) Time to fall in the hindlimb suspension test from the same experimental groups shown in (A). Data represent mean and SEM. Statistics were performed with two-way ANOVA and Bonferroni’s multiple comparison test. Not Significant. (TIF) [file pone.0267990.s003.tif]

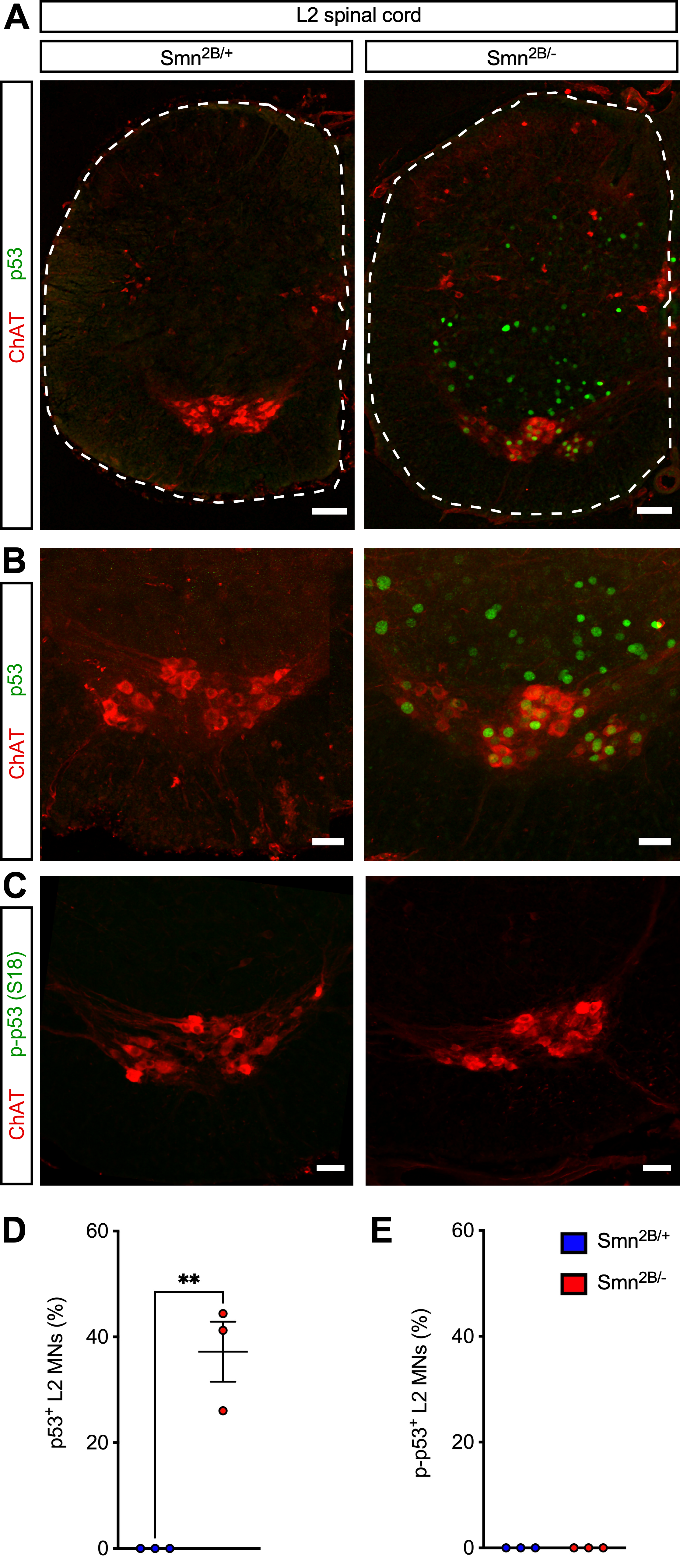

Supplement: S3 Fig — (A) ChAT and p53 immunostaining of the L2 spinal cord from control (Smn2B/+) and SMA (Smn2B/-) mice at P16. Scale bars: 100 μm. (B) ChAT and p53 immunostaining of L2 motor neurons from the same groups as in (A). Scale bars: 50 μm. (C) ChAT and phospho-p53S18 immunostaining of L2 motor neurons from the same groups as in (A). Scale bars: 50 μm. (D) Percentage of p53+ L2 motor neurons from the same groups as in (A). (E) Percentage of phospho-p53S18+ L2 motor neurons from the same groups as in (A). Data represents individual values, mean and SEM from 3 mice per group. Statistics were performed with two-tailed unpaired Student’s t-test. ** P < 0.01. (TIF) [file pone.0267990.s004.tif]

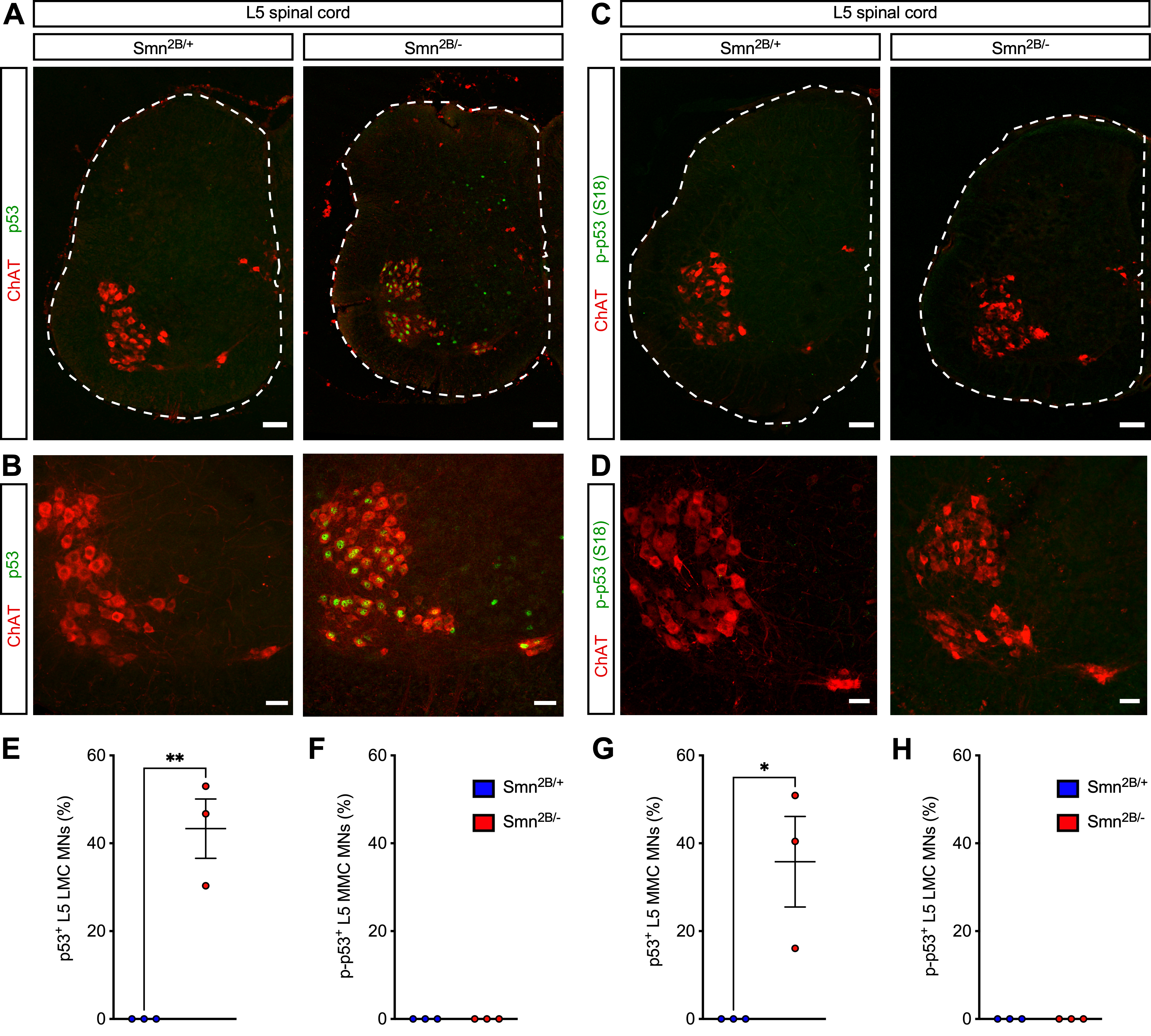

Supplement: S4 Fig — (A) ChAT and p53 immunostaining of the L5 spinal cord from control (Smn 2B/+) and SMA (Smn2B/-) mice at P16. Scale bars: 100 μm. (B) ChAT and p53 immunostaining of L5 motor neurons from the same groups as in (A). Scale bars: 50 μm. (C) ChAT and phospho-p53S18 immunostaining of the L5 spinal cord from the same groups as in (A). Scale bars: 100 μm. (D) ChAT and phospho-p53S18 immunostaining of L5 motor neurons from the same groups as in (A). Scale bars: 50 μm. (E and G) Percentage of p53+ L5 LMC (E) and L5 MMC (G) motor neurons from the same groups as in (A). (F and H) Percentage of phospho-p53S18+ L5 LMC (F) and L5 MMC (H) motor neurons from the same groups as in (A). Data represents individual values, mean and SEM from 3 mice per group. Statistics were performed with two-tailed unpaired Student’s t-test. ** P < 0.01; * P < 0.05. (TIF) [file pone.0267990.s005.tif]

Fig. S1 panel A

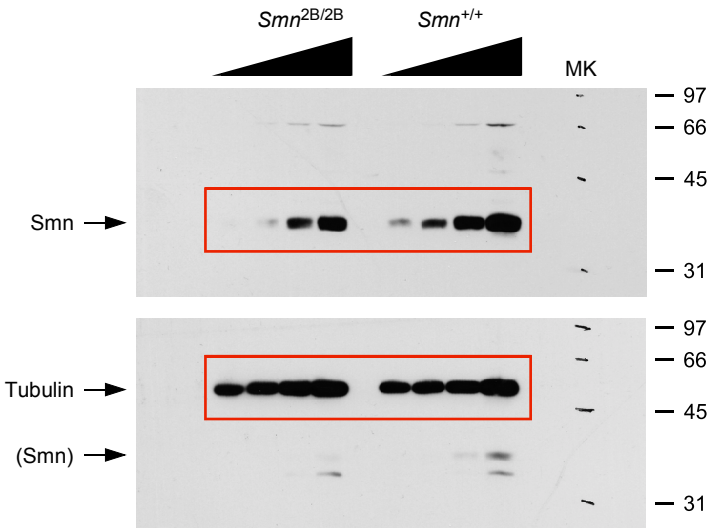

Fig. S1 panel B

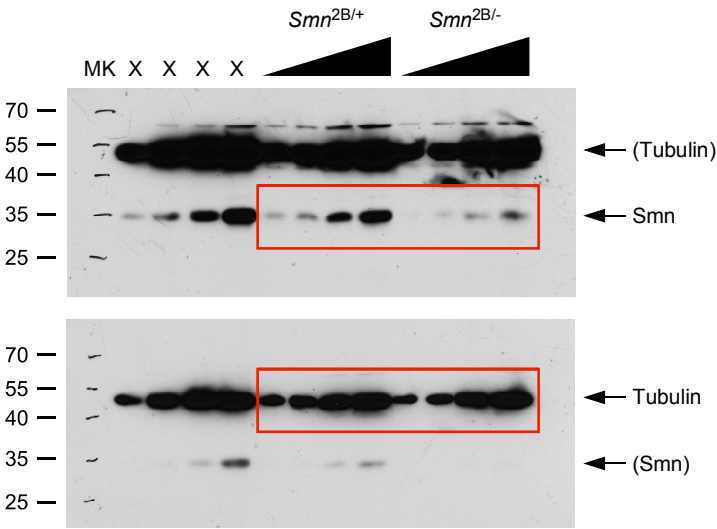

Supplement: S1 Raw images — (PDF) [file pone.0267990.s006.pdf]
